# Experimental and Theoretical Screening of Core Gold Nanoparticles and Their Binding Mechanism to an Anticancer Drug, 2-Thiouracil

**DOI:** 10.3390/molecules29010121

**Published:** 2023-12-24

**Authors:** Génesis Lorenzana-Vázquez, Daniel G. Adams, Lauren G. Reyna, Enrique Meléndez, Ioana E. Pavel

**Affiliations:** 1 Department of Chemistry, University of Puerto Rico, Mayaguez Campus, Mayaguez, PR 00681, USA; genesis.lorenzana@upr.edu; 2 Department of Physical and Environmental Sciences, Texas A&M University—Corpus Christi, Corpus Christi, TX 78412, USAlreyna9@islander.tamucc.edu (L.G.R.)

**Keywords:** Raman spectroscopy, UV-Vis absorption spectrophotometry, gold nanoparticles, 2-thiouracil, SERS, TD-DFT, chemisorption

## Abstract

This study demonstrated the capability of two readily available optical spectroscopy tools, namely UV-Vis absorption spectrophotometry and Raman/surface-enhanced Raman spectroscopy, to select in a rapid and noninvasive manner the most homogenous gold nanoparticle (AuNP) models and to identify their chemical binding mechanism to 2-thiouracil (2-TU). 2-TU is an anticancer drug of great promise in the antiproliferative and photothermal therapies of cancer. The citrate-capped AuNPs emerged as the most stable as well as time- and cost-effective AuNP model out of the three widely used colloidal nanocores (citrate-, borohydride-citrate-, and sodium dodecyl sulfate (SDS)-capped AuNPs) that were examined. 2-TU chemically attached to the relatively monodispersed AuNPs via a chemisorption mechanism. The 2-TU-AuNPs complex formed through the covalent bonding of the S atom of 2-TU to the nanosurface in a vertical orientation. The spectroscopic results were then confirmed with the help of density functional theory (DFT) calculations and other physicochemical characterization tools for nanomaterials such as transmission electron microscopy (TEM), dynamic light scattering (DLS), and zeta potential. Overall, the purified 2-TU-AuNPs were found to be spherical, had an average diameter of 25 ± 2 nm, a narrow size distribution (1–30 nm), a sharp localized surface plasmon resonance (LSPR) peak at 525 nm, and a negative surface charge (−14 mV).

## 1. Introduction

Gold nanoparticles (AuNPs) are biocompatible, can be easily functionalized, and find numerous established or emerging biomedical applications. A few examples include, but are not limited to, imaging, sensing, diagnostics, drug/gene delivery, and cancer therapy [1,2,3,4]. These applications are controlled by the physicochemical properties of AuNPs and their homogeneity. For example, the reduction in particle size below the wavelength (λ) of the incident electromagnetic radiation gives rise to the localized surface plasmon resonance (LSPR) effect in AuNPs. The tunable color of colloidal AuNPs (from violet to wine red) is attributed to the LSPR effect and can be explained by Mie’s theory on the extinction (i.e., absorption and scattering) of light by NPs. Briefly, the LSPR is the result of the collective oscillations of the conduction electrons in AuNPs during their interaction with electromagnetic radiation (i.e., light) [1,2,3,4]. These oscillations give rise to one or multiple absorption maxima in the visible–near-infrared (Vis-NIR) absorption spectra of colloidal AuNPs according to their size and shape. Thus, the LSPR values are key to characterizing and designing versatile AuNPs for biomedical applications. For example, Raman spectroscopy in conjunction with AuNPs can enhance the sensitivity of the Raman detection technique (dσ_R_/dΩ ~ 10^−31^ cm^2^ sr^−1^) down to the single-molecule level (i.e., the surface-enhanced Raman spectroscopy (SERS) effect) by harvesting the LSPR effect [5,6,7].

Raman spectroscopy is a well-established analytical technique that facilitates the rapid and label-free acquisition of vibrational spectra (i.e., molecular “fingerprints”) of an analyte molecule excited with a laser light [5,6,7]. Theory predicted and experiments confirmed that very large increases in Raman cross-sections are associated with target biomolecules located at the nanosurface or nano-sized interstitial sites of aggregates of silver or gold NPs (i.e., the so-called SERS “hot spots”) [5,6,7]. The greatly enhanced signal observed in SERS (~9–12 orders of magnitude) largely arises from the increases in both the incident and the scattered electromagnetic fields associated with the excitation of the LSPR of AuNPs (i.e., the electromagnetic mechanism). Additional enhancements (~3 orders of magnitude) can result from either the charge transfer between the analyte molecules and AuNPs or their chemical complexation (i.e., the chemical enhancement mechanism) [5,6,7].

This study will examine the capability of two well-established spectroscopy techniques, namely ultraviolet-visible (UV-Vis) absorption spectrophotometry and Raman/SERS, to rapidly screen for the most homogenous colloidal AuNPs and to identify their molecular binding mechanism to a functional agent, 2-thiouracil (2-TU), under biocompatible and nondestructive conditions. These optical molecular techniques were selected because of their worldwide availability in industry and research labs, ease of use, and time- and cost-effectiveness [5,6,7]. Furthermore, Raman (without AuNPs) in conjunction with SERS (with AuNPs) can utilize visible lasers of reduced power to avoid photodamage and unwanted fluorescence events. Both techniques require small amounts of samples and minimum-to-no sample preparation. It is expected that the spectral features of the LSPR peaks in the absorption spectra can be quickly correlated to the shape, size, and size distribution of AuNPs, while the Raman/SERS spectra can provide valuable insights into the chemical binding mechanism of 2-TU to AuNPs. In addition, the colloidal AuNPs have extremely high extinction coefficients, and thus can be easily detected and quantified (down to the nM scale) via UV-Vis absorption spectrophotometry [1]. These hypotheses will be confirmed through complementary but more expensive or time-consuming analyses such as transmission electron microscopy (TEM) and dynamic light scattering (DLS) [8]. DLS can be time-consuming for slow dynamics and has low resolution in the identification of closely spaced multimodal size distributions [8,9]. TEM often experiences artifacts from sample preparation (e.g., drying effects of AuNPs), is destructive, and requires highly specialized personnel. In addition, density functional theory (DFT) and time-dependent density functional theory (TD-DFT) calculations will be completed to rapidly validate the experimental data. The focus will be on the simulated absorption spectra of 2-TU before and after complexation to AuNPs, the corresponding HOMO-LUMO gaps, and the possible bonding configurations (2-TU-AuNPs).

To complete the proposed goal, three widely used colloidal models will be synthesized, purified, and concentrated: citrate- [10], borohydride-citrate- [11], and sodium dodecyl sulfate (SDS)-capped AuNPs [12]. For example, the citrate-capped AuNPs will be fabricated in a “bottom-up” approach through the simple reduction of a gold salt, tetrachloroaurate (III) trihydrate, with trisodium citrate, and the subsequent capping with negative citrate ions [1,2]. It is probably the most common synthesis procedure due to its simplicity, low cost, and biocompatible reagents [1,2]. In fact, the citrate stabilizer is one of the major components of the common anticoagulant CPD (citrate, phosphate, and dextrose) for storing human red blood cells. Citrate is followed by other capping agents such as borohydride, polyvinylpyrrolidone (PVP), polyethylene glycol (PEG), and SDS [1,2,3,4]. Once stabilized, the core AuNPs will be functionalized with 2-TU. 2-TU is an anticancer drug of great promise in antiproliferative and photothermal therapies of cancer. In our former work, AuNPs functionalized with 2-TU (2-TU-AuNPs) were found to enhance the antiproliferative activity of 2-TU in MDA-MB-231 breast cancer cells [13]. 2-TU-AuNPs also have the potential to reduce the 2-TU drug concentration and its side effects during cancer treatments. Thus, the binding mechanism of 2-TU to AuNPs plays a key role in their cell delivery and therapeutic activities and could be rigorously characterized through the proposed experimental and theoretical screening methods. Furthermore, the covalent bonding of thiol groups to AuNPs is one of the most common conjugation procedures of bioactive compounds [14].

## 2. Results and Discussion

### 2.1. Characterization of Core Gold Nanoparticles (AuNPs)

**Figure 1:**
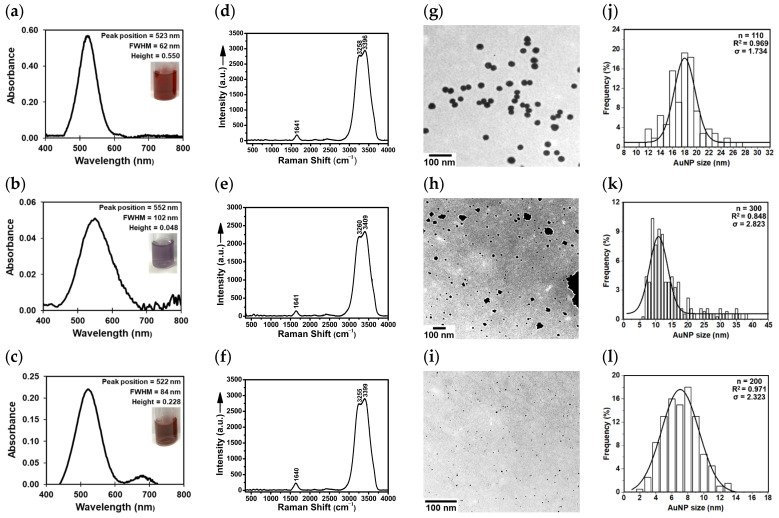
Physicochemical characterization of the core citrate-capped (first row from the top), borohydride-citrate-capped (second row), and sodium dodecyl sulfate (SDS)-capped AuNPs (third row): (**a**–**c**) the baseline-corrected UV-Vis absorption spectra displaying the localized surface plasmon resonance (LSPR) peak of AuNPs (raw data are shown in Figure S1), (**d**–**f**) the SERS spectra demonstrating the chemical purity of the core, colloidal AuNPs with labeled water peaks, (**g**–**i**) TEM images showing the shape and aggregation state of AuNPs, and (**j**–**l**) TEM size histograms showing the size distribution of AuNPs and their Gaussian fitting curves. The spectral features of interest (**a**–**c**) are provided: peak position, peak intensity, and full-width-at-half-maximum (FWHM). The insets (**a**–**c**) show a vial of the AuNP colloid.

Figure 1 displays the representative UV-Vis absorption spectra, Raman spectra, TEM images, and TEM size histograms of the three colloidal AuNP models: citrate-capped, borohydride-citrate-capped, and SDS-capped AuNPs.

The UV-Vis absorption spectra of all core AuNPs (Figure 1a–c) showed an LSPR in the 522–552 nm region depending on their shape, average size, and size distribution [13,15,16,17]. It is well known that the number of the LSPR peaks decreases as the symmetry of the AuNP increases [2,15,16,17]. The presence of a single, relatively symmetrical LSPR peak at the indicated wavelengths is indicative of spherical AuNPs [2,13,15,16,17]. The LSPR peak of the borohydride-citrate-capped AuNPs (552 nm) appeared at a higher wavelength position when compared to the citrate-capped AuNPs (523 nm) and the SDS-capped AuNPs (522 nm). This is indicative of a significantly larger average size for the borohydride-citrate-capped AuNPs when compared to the citrate- and SDS-capped AuNPs. The UV-Vis absorption spectra were collected within minutes with a spectral resolution of ~0.1 nm. In addition, the full-width-at-half-maxima (FWHM) of the LSPR peaks of the borohydride-citrate-capped (102 nm) and SDS-capped AuNPs (84 nm) were significantly larger than the one corresponding to the citrate-capped AuNPs (62 nm). Thus, the citrate-capped AuNPs have a narrower size distribution than the other two core models. The average diameter (*D*) of the colloidal AuNPs can be roughly estimated using the Mie theory (Equation (1)) using the wavelength maximum of the LSPR peak (*λ_LSPR_*), the velocity of the electrons at the Fermi levels (*V_f_* of ~1.4 × 10^6^ m s^−1^), the velocity of light, and the FWHM of the LSPR peak of AuNPs [1,2,3,4]. The theoretical *D* value for the colloidal citrate-capped AuNPs was calculated to be ~6.6 nm, which is smaller than the TEM value estimated below for the AuNPs (20 ± 4 nm). Discrepancies between calculated and experimental *D* values may arise from the drying effects of AuNPs onto TEM grids and the used *V_f_* value. The *V_f_* literature value corresponded to a lattice constant of gold of 0.4 nm (face center cubic for the crystal structure of gold).

(1) $$D=\frac{\lambda_{LSPR}^{2}\cdot V_{f}}{\pi \cdot c\cdot \omega }$$

All colloids appeared red in color except for the borohydride-citrate-capped AuNPs (Figure 1a–c insets), which were purple-greyish due to the larger size distribution and potential aggregation of AuNPs. In good agreement with the literature [18], the SDS-capped AuNPs were found to require extensive ultracentrifugation (e.g., 27,000 rpm for 90 min—Beckman, Brea, CA, USA) for recovery, purification, and further concentration most likely due to their small size. Furthermore, the synthesis of SDS-capped AuNPs (three steps and multiple reagents) was more time and energy consuming than that of the citrate-capped AuNPs (one step and two readily available reagents). Overall, these experimental observations supported the selection of the citrate-capped AuNP candidate and were subsequently confirmed by TEM measurements.

The TEM images (Figure 1g–i) demonstrated that all colloidal AuNPs except for borohydride-citrate-capped-AuNPs are indeed spherical and relatively monodispersed. The citrate-capped AuNPs (1–30 nm) and SDS-capped AuNPs (1–15 nm) had small average diameters of 20 ± 4 nm and 7 ± 2 nm, respectively. The Gaussian fitting [19] of the three TEM size histograms (Figure 1j–l) pointed to a relatively narrow size distribution for both AuNP models and offered additional evidence for their selection, particularly the citrate-capped AuNPs. The citrate-capped AuNPs had a large *R*^2^ factor (0.969) and the smallest *σ* value (1.734). The borohydride-citrate-capped AuNPs exhibited a larger size distribution (1–45 nm) and many aggregated AuNPs (i.e., ~16% of the analyzed *n* = 300 AuNPs) with average diameters > 20 nm. Thus, the borohydride-citrate-capped AuNPs require additional separation steps for the removal of the larger AuNPs or AuNP aggregates and are not suitable candidates for subsequent drug delivery or biosensing studies where homogeneity and size play a key role.

All three colloidal systems were found to be negatively charged with zeta potential values of −38 mV (citrate-capped AuNPs), −14 mV (borohydride-citrate-capped AuNPs), and −37 mV (SDS-capped AuNPs). The highest charges corresponding to better aqueous stability were measured for the citrate-capped and SDS-capped AuNPs. SERS offered rapid (within minutes) proof of the chemical purity of the three colloidal systems (Figure 1d–f). All spectra were dominated by the characteristic Raman bands of water at ~1641 cm^−1^ (bending), 3258 cm^−1,^ and 3396 cm^−1^ (symmetric and asymmetric stretching modes) [20,21]. Additionally, negligible peaks were observed in all three colloidal models if aged longer than one week. This is probably due to the formation of small AuNP aggregates such as dimers or trimers (“hot spots”), which can enhance the ordinary Raman signal of absorbed or nearby citrate ions (e.g., 834, 1378, and 1643 cm^−1^ overlapping with water at 1641 cm^−1^) via the SERS effect [22,23,24]. Citrate ions are known to experience very weak interactions with noble metal NPs, and thus are easily displaced by active functional agents. This makes them a great candidate for subsequent chemical bonding to 2-TU. No significant SERS contributions were noticed from the SDS capping agent [25].

Overall, the results of the UV-Vis absorption, Raman spectroscopy, TEM, DLS, and zeta potential data suggest that the original, citrate-capped AuNPs are the least expensive, fastest, and most simple synthesis leading to a homogenous nanoproduct. The citrate-capped AuNPs have a small average diameter (20 ± 4 nm), narrow size distribution (1–30 nm), small hydrodynamic diameter (32 nm), and minimal aggregation. In our studies, the citrate-capped AuNPs were found to be biocompatible and significantly enhance the antiproliferative activity of 2-TU. These AuNPs functionalized with 2-TU also exhibited photothermal properties during irradiation of breast cancer cells (MDA-MB-231) with green light at 520 nm. More specifically, the half-maximal inhibitory concentration (IC_50_) decreased by a factor of 2 (10.4 nM for AuNPs and 4.4 nM for 2-TU-AuNPs) [13]. Other cancer therapy studies also demonstrate that this type of core citrate-capped AuNPs can efficiently remove the phototoxicity side effects of anticancer drugs such as 6-thioguanine (6-TG) [10]. 6-TG is known to cause an increase in skin cancer incidents in patients undergoing thiopurine treatment [10,11,18]. Furthermore, SDS-AuNPs were reported to severely aggregate in culture media and various buffers, while their toxicity and cellular uptake are poorly characterized [12]. In good agreement with this study, the borohydride-citrate-capped AuNPs had a shelf lifetime of about 1 month, while citrate-capped AuNPs were stable for up to 6 months and had higher concentrations [26].

### 2.2. Purification of Core Gold Nanoparticles (AuNPs)

All core AuNPs were further purified using a 10 kD filter. This filter was associated with a size cutoff of 2–3 nm [20,27]. The UV-Vis absorption spectra of the original and filtered samples (Figure S2) showed that the resulting 10 kD retentate consisted of concentrated AuNPs of similar LSPR profiles, while the 10 kD filtrate was represented mostly by water and residual synthesis reagents (no LSPR peaks). The concentration of the original citrate-capped AuNPs (*c* ~3 nM) was estimated from the Lambert–Beer law (Equation (2)) using a molar extinction coefficient (*ɛ*) of 3.67 × 10^8^ M^−1^ cm^−1^ characteristic to citrate-capped AuNPs with an LSPR peak at ~520 nm and a path length (*l*) of 1 cm [13]. The solvent removal (e.g., 50% by volume) resulted in a corresponding two-fold increase in the absorbance maximum of all AuNPs.

(2) A=ϵ·c·l

**Figure 2:**
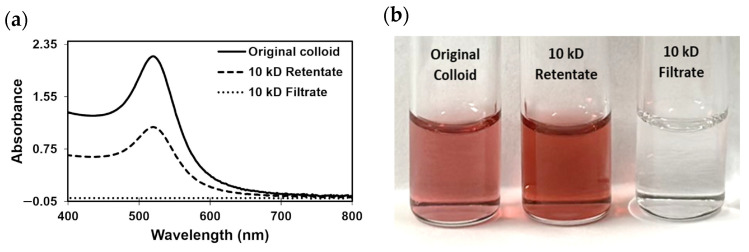
(**a**) UV-Vis absorption spectra and (**b**) a photo of the vials containing citrate-capped colloidal AuNPs before (original) and after 10 kD filtration (10 kD retentate of concentrated AuNPs and 10 kD filtrate of mostly water).

Figure 2a shows an illustrative example of the ~50% increase in the citrate-capped AuNP concentration of the 10 kD retentate (LSPR peak at 523 nm) when compared to the original colloid. This can also be observed visually in Figure 2b, based on the change in color from light red for the original colloid to darker red for the 10 kD retentate of citrate-capped AuNPs. The 10 kD filtrate aliquots were colorless. Further concentration can be achieved as described in our previous work [20]. This method was selected over the gold-standard method of isolation of AuNPs, centrifugation, because it does not cause AuNP aggregation and is more time- and energy-efficient [20].

### 2.3. Experimental Characterization of Gold Nanoparticles Functionalized with 2-Thiouracil (2-TU-AuNPs)

**Figure 3:**
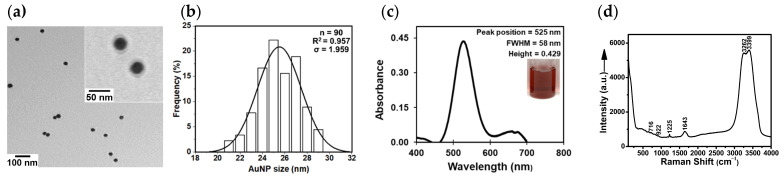
Physicochemical characterization of the core citrate-capped AuNPs functionalized with 2-TU: (**a**) TEM image showing the shape and aggregation state of AuNPs, (**b**) TEM size histogram showing the size distribution of AuNPs and the Gaussian fitting curve, (**c**) baseline-corrected UV-Vis absorption spectrum displaying the localized surface plasmon resonance (LSPR) peak of AuNPs after functionalization, and (**d**) the original SERS spectrum demonstrating the chemical purity of the colloid and the binding of 2-TU to the surface of the citrate-capped AuNPs. The spectral features of interest (**c**) are provided: peak position, peak intensity, and full-width-at-half-maximum (FWHM). The inset (**c**) shows a vial picture of the 2-TU-AuNP colloid.

The filtered, citrate-capped AuNPs were functionalized with 2-TU. The binding of the anticancer drug to the surface of the citrate-capped core AuNPs was confirmed with several physicochemical characterization techniques. First, UV-Vis absorption spectrophotometry demonstrated the surface binding through a red shift of the LSPR peak from 523 nm to 525 nm (Figure 3c). Second, DLS revealed an increase in the hydrodynamic diameter from 32 nm to 45 nm after the functionalization of the core citrate-capped AuNPs with 2-TU, while the surface charge remained overall negative. Third, TEM data further confirmed that these 2-TU-AuNPs were spherical, had an average diameter of 25 ± 2 nm, and a narrow size distribution (1–30 nm) (Figure 3a,b). In addition, a small halo of organic matter (2-TU) was noticed in the TEM image of the 2-TU-AuNPs as a result of the chemical binding of 2-TU to the nanosurface (Figure 3a). Fourth, the SERS spectrum of the 2-TU-AuNPs offered insight into the chemical binding mechanism of 2-TU (Figure 3d).

**Figure 4:**
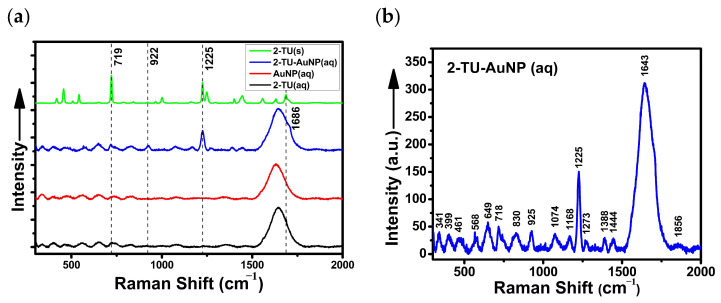

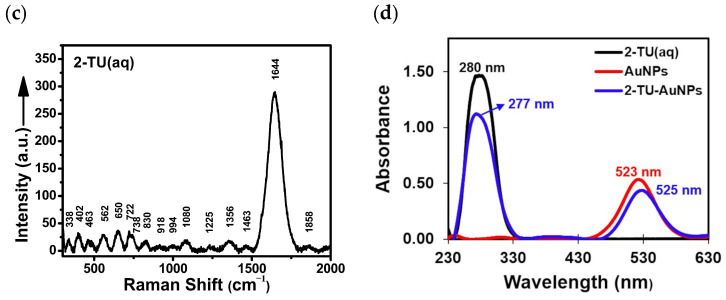
Spectroscopic characterization of the chemical binding of 2-thiouracil (2-TU) to the surface of core citrate-capped AuNPs. Raman (without AuNPs) and SERS (with AuNPs) spectra in the (**a**) 100–2000 cm^−1^ of 2-TU (solid—*s*), 2-TU-AuNPs (aqueous—*aq*), and citrate-capped AuNPs (*aq*) were shifted upwards for comparison reasons with the 2-TU solution (*aq*). (**b**,**c**) Closeup looks at the 300–2000 cm^−1^ fingerprint regions of 2-TU-AuNPs (*aq*) and 2-TU (*aq*), respectively. (**d**) UV-Vis absorption spectra of aqueous (*aq*) samples of 2-TU, AuNPs, and 2-TU-AuNPs in the 230–630 nm fingerprint regions.

**Figure 5:**
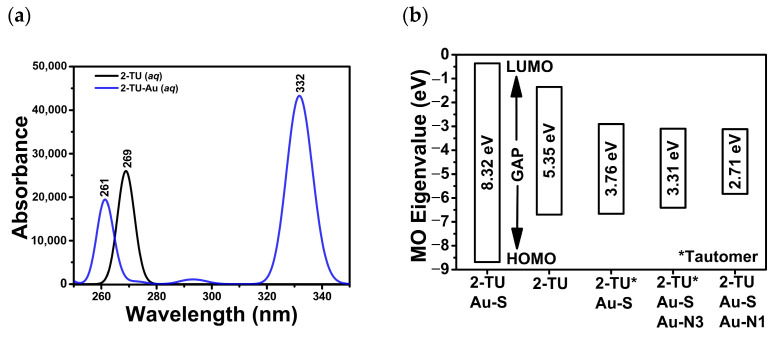
(**a**) Simulated UV-Vis absorption spectra of the free 2-thiouracil (2-TU) and the 2-TU complexed to a single Au atom through the S atom (monodentate Au-S(2) configuration). (**b**) HOMO-LUMO gaps (eV) of the possible bonding configurations of the two tautomeric forms of 2-TU (pH = 6.7) to Au atoms. * Denotes the alternate tautomeric forms with a double bond between N1 and C2 (Figure S3).

In our study, the SERS spectrum of the 2-TU-AuNPs colloid (pH = 6.7) exhibited new peaks when compared to the unfunctionalized, citrate-capped AuNPs (Figure 4a). Although most of these spectral features are small in intensity when compared to the water signals (i.e., 1643, 3262, and 3399 cm^−1^) of the overall 100–4000 cm^−1^ spectrum of 2-TU-AuNPs, they are the strong (e.g., 718 and 925 cm^−1^) and very strong (e.g., 1225 cm^−1^) if excluding the characteristic vibrational modes of water (Figure 4b). These vibrational modes are also present in the Raman spectra of 2-TU powder (719, 922, and 1225 cm^−1^—Figure 4a) and 0.8 mM of 2-TU aqueous solution (pH = 6.3; 722, 918, and 1225 cm^−1^—Figure 4c). However, they are not enhanced in intensity as observed in the SERS spectrum of 2-TU-AuNPs (i.e., 925 and 1225 cm^−1^—Figure 4b). In another SERS study, which was conducted on 0.04 mM of 2-TU (pH = 7) deposited as an analyte onto glass slides covered with citrate-capped AuNPs, these marker bands were observed at similar positions (718, 918, and 1224 cm^−1^) [28]. These studies also showed that pH has no influence on the adsorption behavior of 2-TU (pK_a1_ < 0.5 for uracil [1]) at the AuNPs [28]. The peak at 1224 cm^−1^ was assigned to the C6H and N1H scissoring coupled to the C2N3C4 asymmetric stretchings of 2-TU [28]. The shifts at 718 and 918 cm^−1^ were attributed to the 2-TU ring deformations and N3C4C5 symmetric stretchings [28]. These vibrational modes (e.g., 718, 922, and 1225 cm^−1^) correspond to in-plane vibrations, suggesting vertical or tilted adsorption of 2-TU onto the citrate-capped AuNPs, and the immediate proximity of its S and N atoms to the nanosurface (Figure 5). This binding geometry is supported by the SERS surface selection rules [28,29,30]: the in-plane vibrational modes of 2-TU are more enhanced than its out-of-plane vibrational modes when the drug molecules are adsorbed vertically onto the AuNP nanosurface. Other in-plane vibrational modes were detected at 1388 and 1444 cm^−1^ in the spectrum of 2-TU-AuNPs (Figure 4b). There were also considerable shifts (~4–7 cm^−1^) in the peaks at 718 and 925 cm^−1^ of 2-TU-AuNPs with respect to those of the aqueous solution of 2-TU (Figure 4b,c). The spectral resolution was ~1 cm^−1^. The chemical attachment of 2-TU through its S atom was further substantiated by the appearance of a CS stretching mode [28] at 1168 cm^−1^ in the spectrum of 2-TU-AuNPs (not present or very small for 2-TU (*aq*)). Prior theoretical studies on 2-TU and its chemisorption mechanism demonstrated both 2-TU-AuNP complexed tautomers (Figure S3) as possible at this pH value of 6.7 [28]. Overall, the observed Raman/SERS spectral changes suggest a chemisorption of 2-TU to the AuNPs through the S and/or N atoms, in an inclined or vertical orientation. No peaks characteristic to the Au-S or Au-N stretching modes could be visibly assigned in the low spectral region of the 2-TU-AuNP spectrum due to interference of the strong Rayleigh scattering.

Additional evidence for the proposed covalent binding of 2-TU to the core AuNPs was offered by the UV-Vis absorption spectra. A new absorption maximum appeared at ~277 nm in the 2-TU-AuNP spectrum (Figure 4d). This absorption peak (280 nm) is characteristic of the π-π* electronic transitions of the 2-TU aqueous solution (*aq*) in the pH range of ~2–7 [1,31]. It was not present in the absorption spectrum of core, citrate-capped AuNPs (Figure 4d), indicating the chemical binding of 2-TU to AuNPs through its spectral appearance and 3 nm shift for the 2-TU-AuNP sample.

### 2.4. Theoretical Characterization of 2-Thiouracil (2-TU) and 2-TU-Au Complexes

The simulated, aqueous UV-Vis absorption spectra (Figure 5a) are in good agreement with the experimental data. The theoretical calculations led to an absorption peak at 269 nm for the free 2-TU and 261 nm for the monodentate Au complex bound through the S atom. The 2-TU absorbance value was near the literature value for the non-complexed, aqueous species [1,31]. This absorption maximum experienced a shift of 8 nm upon its complexation to Au atoms (2-TU-Au). A shift to smaller wavelengths was detected experimentally (Figure 4d).

**Figure 6:**
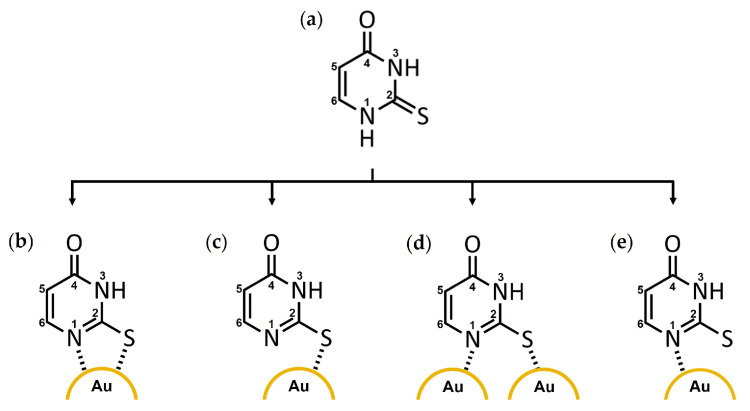
Possible coordination modes of 2-thiouracil (2-TU) to gold nanoparticles (AuNPs): (**a**) 2-TU, (**b**) N(1)-Au-S(2) bidentate, (**c**) Au-S(2) monodentate, (**d**) Au(1)-N(1), Au(2)-S(2), and (**e**) Au-N(1) with labeled atoms.

Calculated HOMO-LUMO energy gaps are generally reliable predictors of the stability of molecular species. Previous studies suggest that time-dependent (TD)-DFT methods are superior to DFT approaches in predicting the energies of the HOMO and LUMO orbitals. However, discrepancies between theoretical and experimental results are expected even with TD-DFT methods. Several relevant 2-TU tautomeric forms (pH = 6.7) were examined in the presence and absence of Au atoms, in the aqueous phase (Figure S3). The HOMO-LUMO energy gap (Figure 5b) was the largest (8.32 eV) for the 2-TU-Au complex bound through the sulfur atom. This theoretical result confirmed the chemisorption mechanism suggested by the Raman/SERS experimental data. The most stable and most probable bonding configuration corresponded to the Au-S(2) monodentate complex (Figure 6c and Figure S3c). The HOMO-LUMO values for the other tautomeric configurations (Figure S3) were 3.76, 3.31, and 2.71 eV.

The gas-phase DFT studies led to similar results: the most likely interaction between AuNPs and 2-TU occurs in an Au-S(2) coordination geometry (Figure 6c). The Au-S(2) bond persisted throughout the optimization, and the resulting bond distance was 2.4 Å, which is indicative of a single Au-S covalent bond. The bidentate N(1)-Au-S(2), the monodentate Au(1)-N(1) and Au(2)-S(2), and monodentate Au-N(1) complexes were also optimized for verification purposes (Figure 6). The N(1)-Au-S(2) structure calculation (Figure 6b) resulted in the cleavage of the Au-N(1) bond, while Au remained coordinated to S(2) and yielded the favored monodentate Au-S(2) structure (Figure 6c). The initial N(1) and S(2) bridge coordination to two different Au atoms (Figure 6d) led to a final complex with Au-Au bonds and no coordination to N(1) and S(2). Thus, this bridge coordination mode is not favorable or is less likely to occur. Similar observations were made for the initial monodentate Au-N(1) complex (Figure 6e) with no final Au-N(1) interactions.

## 3. Materials and Methods

### 3.1. Chemicals

Chloroauric acid trihydrate (HAuCl_4_·3H_2_O, 99.9%), L-ascorbic acid (C_6_H_8_O_6_, ≥99%), sodium borohydride (NaBH_4_, ≥98.0%), and 2-thiouracil (C_4_H_4_N_2_OS, 97%) were purchased from Sigma-Aldrich, St. Louis, MO, USA, and were used without further modifications. Sodium citrate dihydrate (C_6_H_5_Na_3_O_7_·2H_2_O, >95%), dodecyl sulfate (C_12_H_25_NaO_4_S, ≥99%), hydrochloric acid (HCl, 37%), and nitric acid (HNO_3_, 70%) were obtained from Fisher Scientific, Pittsburgh, PA, USA. Ultrapure water (~18 MΩ·cm) was utilized for all syntheses and solution preparations. All glassware and the magnetic stir bars were cleaned with aqua regia solution (HCl: HNO_3_ volumetric ratio of 3:1) and rinsed with ultrapure water before use.

### 3.2. Synthesis of Citrate-Capped AuNPs

The synthesis of citrate-capped AuNPs was performed following a procedure by Wang et al. [10]. In this one-step synthesis, 143 mL of 0.28 mM of chloroauric acid aqueous solution was brought to boiling, and 7 mL of 38.8 mM of sodium citrate was added. The resulting mixture was stirred for 15 min, at 350 rpm, and was then cooled down to room temperature, with additional stirring for 1 h.

### 3.3. Synthesis of Borohydride-Citrate-Capped AuNPs

The synthesis of borohydride-citrate-capped AuNPs was conducted using a modified procedure by Karimi-Maleh et al., at room temperature [11]. In the first step, 0.5 mL of 25.4 mM of chloroauric acid solution was diluted in 50 mL of distilled water, and 0.5 mL of 3.4 mM sodium citrate solution was added to this solution (~1 min stirring). In the second step, 0.5 mL of 19.8 mM of sodium borohydride solution was added as a reducing agent, and the resulting mixture was stirred for ~10 min.

### 3.4. Synthesis of Sodium Dodecyl Sulfate (SDS)-Capped AuNPs

SDS-capped AuNPs were synthesized using a modified procedure by Li et al. at room temperature [12]. In the first step, Au seeds were prepared by reducing 20 mL of 0.25 mM of chloroauric acid solution with 0.6 mL of 0.1 M of sodium borohydride solution. Both solutions contained 25 mM of sodium citrate. In the second step, 1 mL of Au seed solution was added to 9 mL of 0.25 mM of chloroauric acid solution containing 1 mM of SDS, and the mixture was magnetically stirred at 1250 rpm for 10 min. In the third step, 0.1 mL of 0.05 M of L-ascorbic acid solution was added drop by drop, and the mixture was stirred for 10 min.

### 3.5. Purification of AuNPs

Batches of colloidal AuNPs (10 mL) were purified through tangential flow filtration (TFF) using a 10 kD hollow fiber filter (Repligen Corp., Rancho Dominguez, CA, USA) according to a previously described procedure [20]. In this purification process, colloidal AuNPs that were smaller than the pore size of the filter (i.e., the filtrate) passed through together with any excess reagents, byproducts, and the water solvent, while colloidal AuNPs larger than the pore size (i.e., the retentate) were swept along by the tangential flow and recirculated as needed for further purification and concentration. The final, purified retentate of AuNPs (5 mL) was collected and redispersed in an equal volume of deionized water as needed for further studies.

### 3.6. Functionalization of AuNPs with 2-Thiouracil (2-TU)

The functionalization of AuNPs was conducted using a modified procedure by E. Iglesias [1]. Briefly, 2 mL of 0.8 mM of 2-TU aqueous solution was added to 20 mL of the purified citrate-capped AuNPs with continuous stirring at 350 rpm for 30 min.

### 3.7. Experimental Characterization of Unfunctionalized and Functionalized AuNPs

The UV-Vis absorption spectra (200–800 nm) of the colloidal AuNPs were collected with the help of a Cary 50 dual-beam UV-Vis spectrophotometer (Agilent Inc., Santa Clara, CA, USA) in quartz cuvettes of a 1-cm path length, and at a spectral resolution of ~0.1 and ~1.0 nm. Original spectra (Figure S1) were baseline-corrected using a B-spline function, and spectral features of interest (peak intensity, full-width-at-half-maximum, and peak position) were established for the localized surface plasmon resonance peak (LSPR). The colloidal AuNPs were imaged using a 208S transmission electron microscope (TEM, Philips) at an accelerating potential of 70 kV. Minute volumes of colloidal samples (5 μL) were deposited on 300-mesh copper grids (Electron Microscopy Sciences, Hartfield, PA, USA) coated with a carbon support film and were air-dried before imaging with a high-resolution Gatan Bioscan camera. The ImageJ 1.46R software was utilized to process the micrograph images for the average size and size distribution of AuNPs. Dynamic light scattering (DLS) and zeta potential measurements of AuNPs were carried out with a Zetasizer Nano ZS90 system. Colloidal AuNPs (0.75–1.0 mL) were placed in disposable polystyrene cuvettes and were measured at a temperature of 25 °C using a laser source wavelength of 532 nm over 12 cycles. The acquisition parameters were as follows: an equilibration time of 120 s for each sample, a dispersant (water) refractive index of 1.330, a viscosity of 0.8872 cP, and a dispersant dielectric constant of 78.5. Raman spectra (100–4000 cm^−1^) were collected using a LabRam HR 800 system (Horiba Inc., Piscataway, NJ, USA) equipped with a high-resolution confocal Raman microscope (high-stability BX41) and Olympus objectives (50× and 100× long working distance). The samples were excited with a HeNe laser (22 mW output) and the backscattered signal was recorded using a thermoelectric open-electrode CCD camera (1024 × 256 pixels) and the LabSpec 5 software. The holographic grating was 600 grooves mm^−1^, and the confocal hole was set at 300 µm. These acquisition parameters resulted in a spectral and spatial resolution of ~1 cm^−1^ and ~0.5–1.0 µm (100×). Liquid samples were measured in quartz cuvettes, while solid samples were placed onto precleaned glass microscope slides.

### 3.8. Theoretical Characterization of 2-TU and 2-TU-Au Complexes

A density functional theory (DFT) study was performed using the Gaussian 09 software [32] to elucidate the possible coordination modes of 2-TU to AuNPs. The 2-TU structure was first optimized in the gas phase using the B3LYP density hybrid function and the 3–21G basis set. The coordination modes of the optimized 2-TU structure to Au atoms were studied using the B3LYP function and the LanL2DZ basis set. Four initial structures were submitted for optimization based on the experimental data and literature (Figure 6).

Molecular models of free 2-TU and various configurations of 2-TU-Au complexes were also developed and auto-optimized using the open-source software Avogadro 1.2.0 [33]. The resulting molecular geometries were further optimized using the open-source software Orca 5.0.4 to simulate the absorption spectra of the free 2-TU and complexed 2-TU-Au species [34]. The PBE0 hybrid functional and the D3BJ dispersion correction were used in conjunction with the RIJCOSX approximation to account for the solvent effect of the aqueous solution using the conductor-like polarizable continuum model (CPCM) [35,36]. Time-dependent density functional theory (TD-DFT) calculations were run in Orca and used to generate simulated UV-Vis absorption spectra of free 2-TU and the 2-TU-Au complexes [37,38,39]. Eigenvalues of frontier molecular orbitals (FMOs) from self-consistent field (ΔSCF) calculations were then used to calculate the HOMO-LUMO energy gaps of the free 2-TU and the 2-TU-Au complexes [40,41,42]. This was accomplished by subtracting the LUMO energy value from the HOMO value for the explored coordination modes (Figure S3).

## 4. Conclusions

Raman/SERS and UV-Vis absorption spectrophotometry facilitated the rapid (within minutes) collection of data from aqueous colloidal AuNPs with no sample preparation or photodamage (directly into cuvettes). The analyses of the spectroscopic data enabled the simple selection of the most homogenous and time- and cost-effective AuNP core for subsequent functionalization with the anticancer drug, 2-TU, and the identification of the chemisorption mechanism of 2-TU to the nanosurface. The physicochemical characterization results of the unfunctionalized AuNPs and functionalized 2-TU-AuNPs were then confirmed using theoretical calculations (DFT and TD-DFT) and other physicochemical characterization techniques (TEM, DLS, and zeta potential) on the same samples (nondestructive). The overall results demonstrated the capability of these optical molecular spectroscopy tools and open-source software (Avogadro and Orca) in aiding with the rapid screening of potential core AuNP candidates for subsequent anticancer drug functionalization and their chemical binding mechanism. This combination of experimental and theoretical tools could be applied to a broad spectrum of anticancer drugs and other functional agents/linkers that encompass chemical moieties of high chemical affinity to the gold nanosurface (e.g., aromatic rings, thiol, or amine groups with lone pairs of electrons).

According to the American Cancer Society, approximately 1.9 million new cases of diagnosed cancer and over 609,000 cancer-related deaths are expected in 2023 in the U.S. [43]. Cancer therapies are mostly limited to surgery, chemotherapy, and radiotherapy. Cancer treatments are planned based on the cancer type and its advancement. One of the major side effects of radiotherapy and chemotherapy, namely normal tissue toxicity, can be reduced or removed with the help of AuNP-based therapeutics [44,45]. In chemotherapy, AuNPs functionalized with anticancer drugs can enhance their intracellular uptake and antiproliferative activity. In radiotherapy, AuNPs-based radiosensitizers can improve the local radiation dose and minimize damage to surrounding health tissues. Currently, there are over 20 nano-based therapeutics approved for clinical use [45], and 2-TU-AuNPs showed great promise as both antiproliferative and photothermal therapeutic agents in our preliminary work on MDA-MB-231 breast cancer cells [13].

## Data Availability

Data are contained within the article and Supplementary Materials.

## Supplementary Materials

The following supporting information can be downloaded at: https://www.mdpi.com/article/10.3390/molecules29010121/s1, Figures S1 and S2: UV-Vis absorption characterization data of colloidal AuNPs before and after 10-kD filtration. Figure S3: 2-TU-Au coordination configurations for the theoretical simulations of UV-Vis absorption spectra.
