# Supplementary material for: Experimental and Theoretical Screening of Core Gold Nanoparticles and Their Binding Mechanism to an Anticancer Drug, 2-Thiouracil
Source: Molecules. 2023 Dec 24;29(1):121. doi: 10.3390/molecules29010121 (PMC10779594; doi:10.3390/molecules29010121)
Supplement: Supplementary file 1 [file molecules-29-00121-s001.zip › molecules-2721807-supplementary.pdf]

Supplementary Information

Experimental and theoretical screening of core gold nanoparticles and their binding mechanism to an anticancer drug, 2-thiouracil

Génesis Lorenzana-Vázquez [1], Daniel G. Adams [2], Lauren G. Reyna[2], Enrique Meléndez [1,*], and Ioana E. Pavel [2,*]

Figure S1 displays the raw UV-Vis absorption data, which were collected on the core AuNP models before filtration.

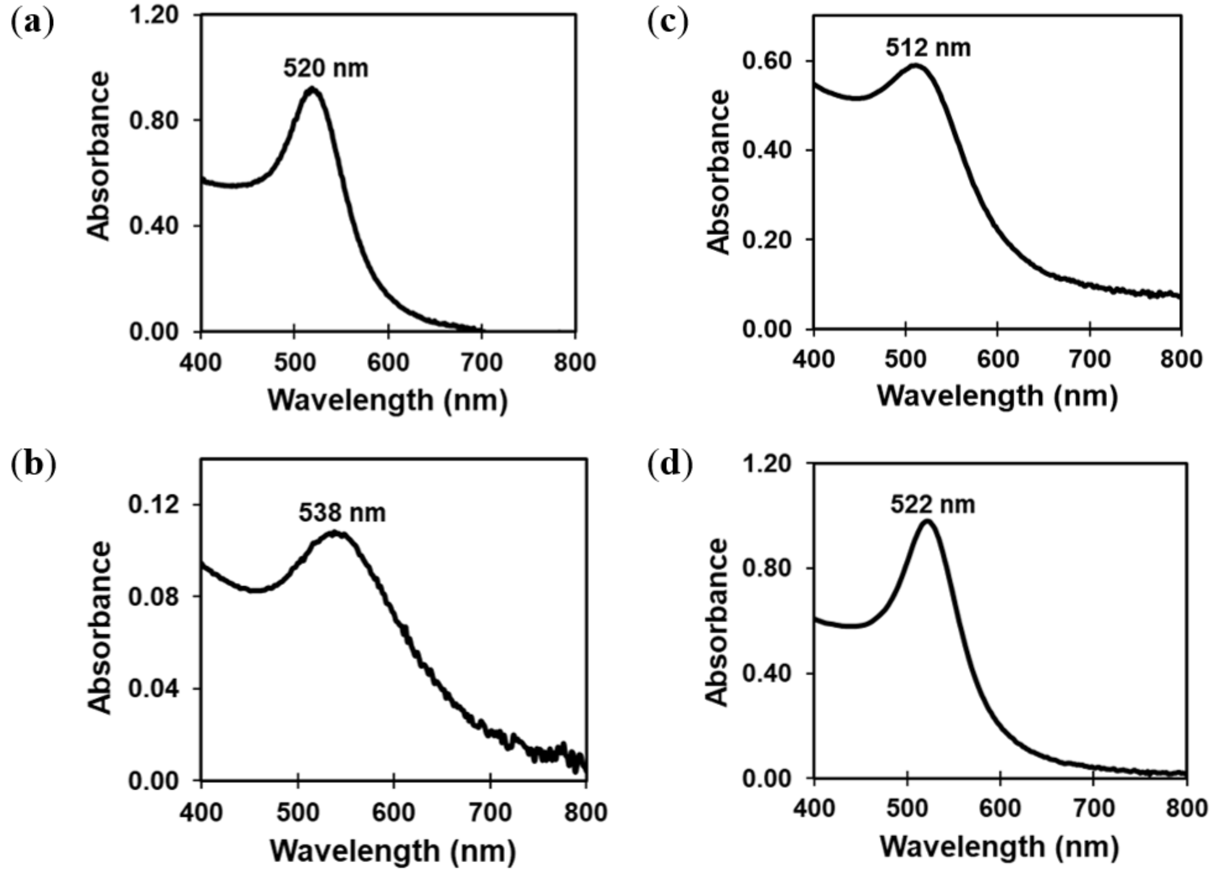

Figure S1. Original UV-Vis absorption spectra of (**a**) citrate-capped, (**b**) borohydride-citrate-capped, (**c**) sodium dodecyl sulfate (SDS)-capped AuNPs, and (**d**) citrate-capped AuNPs functionalized with 2-TU in the 400-800 nm spectral range.

Figure S2 displays the raw UV-Vis absorption data and the pictures of the vials containing colloidal aliquots, which were collected on the original, core AuNPs, before and after filtration.

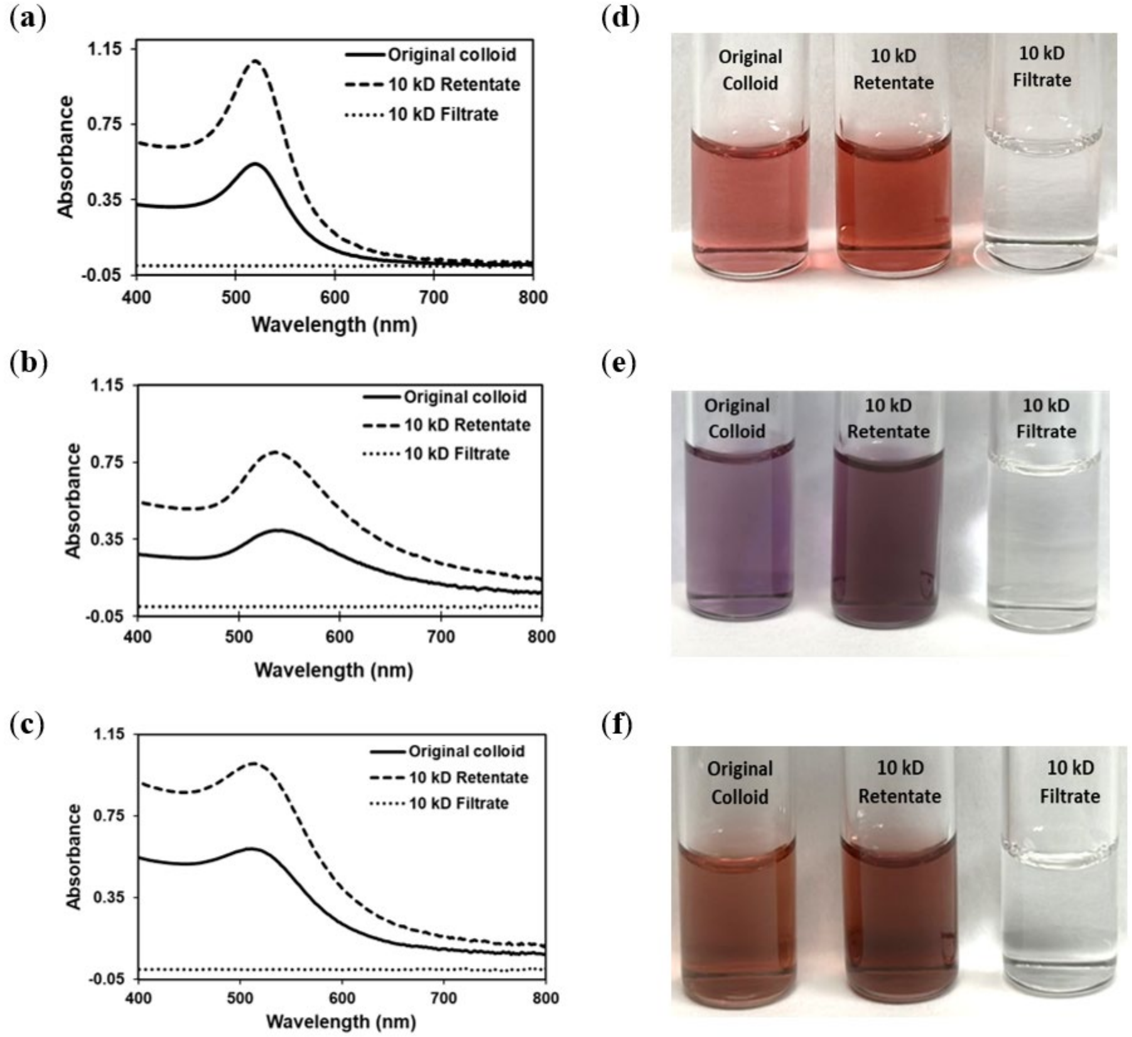

Figure S2. Physicochemical characterization of the colloidal AuNPs before and after the 10-kD filtration: (**a, d**) citrate-capped AuNPs, (**b, e**) borohydride-citrate-capped AuNPs, and (**c, f**) SDS-capped AuNPs. Panels (**a-c**) show the corresponding UV-Vis absorption spectra, while panels (**d-f**) represent images of the vials containing the original colloid, the 10-kD retentate containing concentrated AuNPs, and the 10-kD filtrate consisting mostly of water.

Figure S3 shows the labeled structures of 2-TU and 2-TU-Au complex configurations that were input into Orca for the theoretical simulations of the UV-Vis absorption spectra.

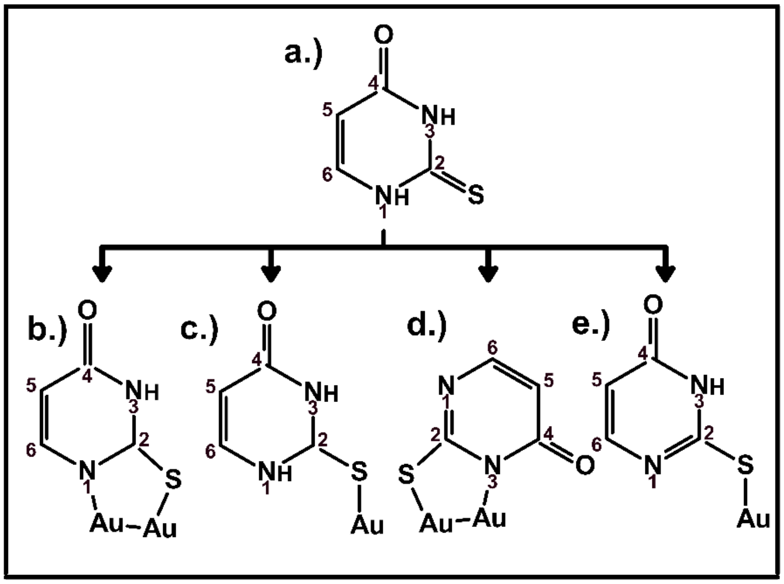

Figure S3. Labeled structures of (**a**) free 2-TU and (**b-e**) possible bonding configurations of the two tautomeric forms of 2-TU (pH = 6.7) complexed to Au atoms. The optimized structures (**a**) and (**c**) were utilized in the theoretical simulations of the UV-Vis absorption spectra.
